# Diagnostic ureteroscopy for upper tract urothelial carcinoma: friend or foe?

**DOI:** 10.1080/2090598X.2021.1883810

**Published:** 2021-02-16

**Authors:** Angelo Territo, Andrea Gallioli, Iacopo Meneghetti, Matteo Fontana, Jordi Huguet, Joan Palou, Alberto Breda

**Affiliations:** Urology Department, Fundació Puigvert, Autonomous University of Barcelona, Barcelona, Spain

**Keywords:** Diagnostic biopsy, diagnosis, radical nephroureterectomy, upper tract urothelial carcinoma, ureteroscopy

## Abstract

**Introduction:**

The European Association of Urology guidelines recommend offering kidney-sparing surgery (KSS) as a primary treatment option to patients with low-risk tumours. Cystoscopy, urinary cytology, and computed tomography urography (CTU) do not always allow correct disease staging and grading, and sometimes there is even a lack of certainty regarding the diagnosis of UTUC. Diagnostic ureteroscopy (d-URS) may therefore be of crucial importance within the diagnostic framework and fundamental in establishing the appropriate therapeutic approach.

**Evidence acquisition and synthesis:**

A systematic review of the literature was performed in accordance with the Preferred Reporting Items for Systematic Reviews and Meta-Analyses (PRISMA) statement. Risk of bias was assessed using Risk of Bias in Non-randomized Studies of interventions (ROBINS-I). Overall, from 3791 identified records, 186 full-text articles were assessed for eligibility. Finally, after a quantitative review of the selected literature, with the full agreement of all authors, 62 studies were considered relevant for this review.

**Results:**

CTU has a sensitivity and specificity for UTUC of 92% and 95% respectively, but is not able to detect small or flat lesions with adequate accuracy. The sensitivity of voided urinary cytology for UTUC is around 67–76% and ranges from 43% to 78% for selective ureteric urine collection. As no technique offers a diagnosis of certainty, d-URS can allow an increase in diagnostic accuracy. In the present review the pros and cons of d-URS were analysed. This technique may provide additional information in the selection of patients suitable for neoadjuvant chemotherapy or KSS, distinguishing between normal tissue and low- and high-grade UTUC thanks to the emerging technologies.

**Conclusions:**

Information obtainable from d-URS and ureteroscopic-guided biopsy can prove extremely valuable when the diagnosis of UTUC is doubtful or KSS is being considered. Notwithstanding concerns remain regarding the potential risk of bladder recurrence, cancer dissemination, and/or delay in radical treatment.

**Abbreviations:** CLE: confocal laser endomicroscopy; CSS: cancer-specific survival; CTU: CT urography; d-URS: diagnostic ureteroscopy; EAU: European Association of Urology; HR: hazard ratio; IMAGE1S: Storz professional imaging enhancement system; IVR: intravesical recurrence; KSS: kidney-sparing surgery; MFS: Metastasis-free survival; NAC: neoadjuvant chemotherapy; NBI: narrow-band imaging; OCT: optical coherence tomography; RFS: Recurrence-free survival; RNU: radical nephroureterectomy; ROBINS-I: Risk of Bias in Non-randomized Studies of interventions; URS(-GB): Ureteroscopy(-guided biopsy); UTUC: upper tract urothelial carcinoma; UUT: upper urinary tract

## Introduction

Upper tract urothelial carcinoma (UTUC) accounts for 5–10% of urothelial carcinomas [1]. To date, radical nephroureterectomy (RNU) with bladder cuff excision still represents the ‘gold standard’ treatment for UTUC. Nevertheless, the European Association of Urology (EAU) guidelines recommend offering kidney-sparing surgery (KSS) as a primary treatment option to patients with low-risk tumours [1,2]. The availability of preoperative predictive tools that yield reliable information on tumour location, size, stage, and grade and any tumour-associated conditions is of fundamental importance in guiding the selection of candidates for such treatment. The EAU guidelines make a Grade-A recommendation for the performance of cystoscopy, urinary cytology, and CT urography (CTU) in the diagnostic evaluation of UTUC. They also advise that diagnostic ureteroscopy (d-URS) and biopsy should be performed in cases in which additional information may have an impact on treatment decisions [1]. In our opinion d-URS represents an extremely useful tool that guarantees the possibility of carrying out a visual study of the conditions of the upper urinary tract (UUT). This means, for instance, identifying the actual number and size of the lesions. It also represents an irreplaceable tool to seek for and possibly biopsy flat lesions not identifiable on the CTU. The possibility of studying the UUT endoscopically is also of fundamental importance to design or evaluate the feasibility of a conservative approach, especially in those conditions in which the lesion is difficult to reach or localise (i.e. lower calyx). Imaging and optical diagnostic techniques and new devices for performing biopsies represent an extraordinary tool for increasing diagnostic accuracy.

However, the procedure is challenging so that not always achieving optimal results. It is then necessary to carry out a risk stratification of patients to reduce costs, complications, and potential risk of bladder recurrence, cancer dissemination, or delay in radical treatment.

A systematic review of the literature was performed with the aim of taking stock of the current knowledge of the strengths and weaknesses of d-URS in UTUC.

## Evidence acquisition and synthesis

A comprehensive literature search was performed in May 2020 using the PubMed/MEDLINE database using the following terms: ‘upper tract urothelial carcinoma’ OR ‘UTUC’ OR ‘upper urinary tract’ AND ‘biopsy’ OR ‘bladder recurrence’ OR ‘complications’ OR ‘delay’ OR ‘diagnosis’ OR ‘diagnostic ureteroscopy’ OR ‘confocal laser endomicroscopy’ OR ‘grade’ OR ‘IMAGE1S’ OR ‘imaging’ OR ‘narrow-band imaging’ OR ‘neoadjuvant chemotherapy’ OR ‘optical coherence tomography’ OR ‘stage’.

Publications were filtered for English language and full-text availability and included original articles, clinical trials, case reports, meta-analyses, professional society guidelines, and reviews. Letters to the editor, replies, textbooks, and contributions written in languages other than English were excluded from our research. No filters were applied for the publication date.

**Figure 1.**
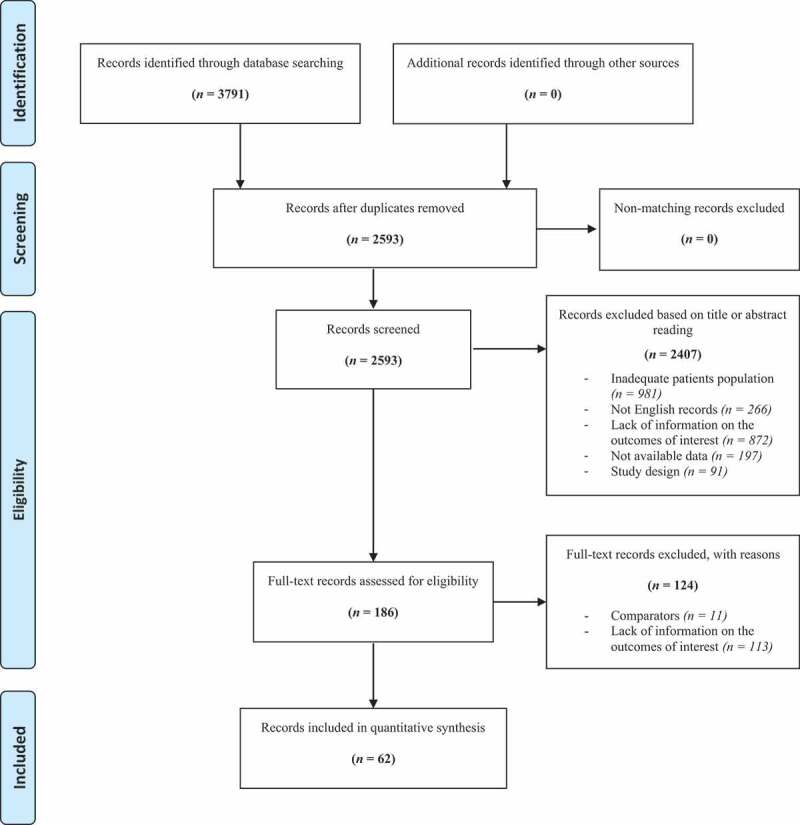
PRISMA diagram for study selection

Eligible articles were screened according to the Preferred Reporting Items for Systematic Reviews and Meta-Analysis (PRISMA) criteria [3] (Figure 1).

Studies were not excluded a priori based on quality reporting assessment. Because only non-randomised studies were retrieved, we adopted the Risk of Bias in Non-randomized Studies of interventions (ROBINS-I) tool to assess the risk of bias in the included studies for the following domains: confounding bias, selection of participants bias, bias in classification of interventions, bias due to deviation from intended interventions, bias due to missing data, the bias in the measurement of outcomes and bias in the selection of the reported results [4]. We used the ROBINS-I tool to evaluate the risk of bias among the studies included as one of the four levels (low, moderate, serious and critical). The results of our risk of bias assessment for all individual studies are shown in Table 1 [7,19–21,25–27,30,32–36,38–40,42,43,46–48,55–58,62,63]. The most problematic domains involved uncontrolled, single-arm studies, small sample sizes, outcomes measurement, and confounding.

**Table 1.** Risk of bias assessment using ROBINS-I

Reference	Bias due to cofounding	Bias in selection of participants into the study	Bias in classification of interventions	Bias due to deviations from intended intervention	Bias due to missing data	Bias in measurement of outcomes	Bias in selection of the reported results	Overall bias
Kleinmann et al., 2013 [19] Lama et al., 2018 [21] Breda et al., 2019 [20] Golan et al., 2015 [25] Tsivian et al., 2014 [26] Gallioli et al.,2020 [7] Lane et al., 2010 [27] Porten et al., 2014 [30] Traxer et al., 2011 [32] Emiliani et al., 2017 [33] Bus et al., 2016 [34] Bui et al., 2015 [35] Breda et al., 2018 [36] Traxer et al., 2013 [42] Schoenthaler et al., 2014 [43] Whitehurst et al., 2017 [38] Somani et al., 2017 [39] Baş et al., 2017 [40] Hendin et al., 1994 [46] Ishikawa et al., 2010 [47] Gurbuz et al., 2011 [48] Lee et al., 2018 [54] Sung et al., 2015 [55] Lee et al., 2016 [56] Yoo et al., 2017 [57] Nison et al., 2013 [61] Boorjian et al., 2005 [62]	Critical Critical Critical Critical Critical Serious Critical Critical Serious Moderate Moderate Moderate Moderate Moderate Moderate Moderate Moderate Moderate Critical Critical Critical Critical Critical Critical Critical Critical Critical	Serious Serious Moderate Serious Serious Moderate Critical Critical Serious Moderate Low Low Low Moderate Low Low Low Low Serious Serious Serious Serious Serious Serious Serious Critical Critical	Moderate Moderate Moderate Moderate Moderate Moderate Serious Serious Moderate Moderate Low Low Low Low Low Low Low Low Moderate Moderate Moderate Moderate Moderate Moderate Moderate Moderate Moderate	Moderate Moderate Moderate Moderate Moderate Moderate Serious Serious Moderate Low Low Low Low No information No information No information No information No information Moderate Serious Serious Serious Serious Serious Serious Serious Serious	Serious Moderate Moderate Critical No information Low Moderate Moderate Low Low Low Low Low Moderate Moderate Moderate Moderate Moderate No information Serious Moderate Moderate Moderate Moderate Moderate Serious No information	Moderate Moderate Moderate Moderate Moderate Moderate Serious Serious Moderate Ciritcal Serious Serious Serious Moderate Moderate Moderate Moderate Moderate Serious Moderate Moderate Moderate Moderate Moderate Moderate Moderate Moderate	Moderate Moderate Moderate Moderate Moderate Low Moderate Moderate Low Serious Low Low Low Moderate Critical Critical Critical Critical Critical Serious Serious Serious Serious Serious Serious Serious Serious	Serious Serious Serious Serious Serious Moderate Critical Critical Moderate Serious Moderate Moderate Moderate Moderate Moderate Moderate Moderate Moderate Serious Serious Serious Serious Serious Serious Serious Critical Critical

The reported studies had moderate, serious, or critical risk of bias. When using the ROBINS-I assessment, it is recommended to exclude studies with a high-risk of bias from analyses. Nevertheless, considering the rarity of the disease and the various technical details regarding diagnosis and therapy analysed, despite the overall high risk of bias, all the studies have been retained for the purposes of the present review. Moreover, we performed many subgroup analyses to investigate so that it could be a source of further heterogeneity.

Overall, from 3791 identified records, 186 full-text original articles were assessed for eligibility. Finally, 62 records were considered relevant for the present review.

## Results

The findings reported in the present review relate to the pros of d-URS, such as histological characterisation of UTUC in select patients eligible for endoscopic treatment and/or neoadjuvant chemotherapy (NAC). The cons of d-URS, i.e. complications, inconclusive diagnosis, risk of cancer dissemination and bladder recurrence, and potential delay in treatment could also affect the prognosis.

### Pros

#### Histopathological confirmation of disease

CTU has the highest diagnostic accuracy among the available imaging techniques, with a sensitivity and specificity of 92% and 95%, respectively [5]. However, CTU may not be able to reliably estimate the actual three-dimensional extension of the disease or to detect small or flat lesions with adequate accuracy, especially in the case of ureteric localisation [6], thus potentially leading to misclassification and undertreatment [7]. The sensitivity of voided urinary cytology for UTUC is around 67–76% and ranges from 43% to 78% for selective ureteric urine collection, being higher for high-grade UTUC [8,9]. Barbotage cytology detects up to 91% of cancers, being as effective as biopsy histology [10].

A d-URS allows exploration of the UUT and is crucial when diagnostic uncertainty exists and in patients who can benefit from KSS [1,11]. A d-URS has the advantage to offer a direct view of the tumour for biopsy and to facilitate selective ureteric sampling for cytology in situ [1,11]. Indeed, *in situ* collection of urine through the barbotage technique has proven to be sensitive if correctly executed, with better diagnostic performance than that of voided urine cytology [12]. It also enables clarification of the nature of non-specific CTU findings, such as wall thickening, and to re-classify suspicious lesions as benign [13].

Grahn et al. [14] compared the performance of CTU and visual assessment during d-URS in 148 patients with suspected UTUC. The d-URS showed a similar sensitivity (89% vs 84%; *P* = 0.36), but greater specificity (51% vs 85%; *P* < 0.001) and accuracy (74% vs 84%; *P* = 0.04) than CTU.

Gallioli et al. [7] found that, among patients with positive CTU and d-URS, the lesions differed in dimensions, number or site in 45/107 (42.1%) cases.

#### Grading, staging, and biopsy for histopathology

Ureteroscopic-guided biopsy (URS-GB) correctly determines tumour grade in most cases, but stage assessment using URS-GB is notoriously difficult.

Subiela et al. [15] conducted a systematic review and meta-analysis including 23 studies on the diagnostic accuracy of URS-GB in predicting stage and grade at final pathology. The authors reported a grade concordance between URS-GB and final pathology of 66% (95% CI 55–77%) for low-grade tumours and 97% (95% CI 94–98%) for high-grade tumours. They also found that identification of high-grade and subepithelial invasion at URS-GB had a moderate (positive predictive value [PPV] 60%, 95% CI 54–66%) and strong correlation (PPV 94%, 95% CI 84–100%) with invasive UTUC at final pathology, respectively. Notwithstanding, undergrading and understaging rates at prior URS-GB compared with final pathology were found in 32% (95% CI 25–38%) and 46% (95% CI 38–54%) of cases, respectively.

It is evident that, to date, the accuracy of URS-GB has been limited by several factors, including insufficient tissue quality and crush artefacts; even if there is no unanimity over whether low biopsy volume in URS-GB affects tumour biopsy grading. Some authors try to overcome these limitations by describing detailed methods for URS inspection and URS-GB, while others have sought to find the best device to obtain an adequate biopsy sample.

In 1997, Tawfiek et al. [16] described in detail their technique to inspect the UUT and perform URS-GB for handling and processing the samples. In a series of 45 patients, Guarnizo et al. [17] proposed a multi-biopsy approach that was able to achieve a diagnostically accurate diagnosis of UTUC in 89% of cases.

Golan et al. [18] proposed a technique to achieve maximal tissue preservation in which the specimen was mounted on filter paper prior to embedding in paraffin.

With the current diagnostic devices, an ideal method to procure adequate tissue sampling is still missing. Basket and 3-F cup biopsy forceps represent the two most commonly used biopsy tools [19]. Basket devices can be front loaded and allow the ensnarement of a piece or even the whole specimen from its base. Thanks to their flexibility they are also more likely to reach those lesions localised in calyces that require considerable deflection of the instrument. Conversely, flat lesions could not be sampled with this tool. Larger diameter forceps can capture large specimens and also flat lesions, but they need to be introduced in the ureteroscope by backloading as the tip of the device is not compatible with the diameter of the working channel. Unfortunately, they bestow stiffness to the flexible ureteroscope, so that sampling of lesions could be impossible if deflection is needed. Furthermore, they require the use of a double lumen ureteroscope or the placement of ureteric access sheath to allow the insertion of the ureteroscope because of the consequent lack of irrigation fluid [20,21] (Table 2 [19–21]).

**Table 2.** Overview of biopsy devices during operative ureteroscopies used in UTUC in the included studies

Reference	Patients treated, *n*	Devices	Successful histology characterisation, *n/N* (%)	Unsuccessful histology characterisation, *n/N* (%)	Grade, *n/N* (%)	Stage, *n/N* (%)	Conclusion
Kleinmann et al., 2013 [19]	127 (303 biopsies)	3-F forceps 2.4-F flat wire basket	3-F forceps 150/237 (63.3) 2.4-F flat wire basket 62/66 (93.9)	3-F forceps 87/91 (95.6) 2.4-F flat wire basket 4/91 (4.4)	3-F forceps specified (80) 2.4-F flat wire basket specified (93)	_	The flat wire basket was superior in achieving pathological diagnosis and in determining tumour grade compared with the 3-F forceps. The forceps were useful for small and sessile lesions.
Lama et al., 2018 [21]	145 (182 biopsies)	3-F forceps BIGopsy forceps 2.4-F nitinol basket	3-F forceps 31/39 (79.5) BIGopsy forceps 64/71 (90.1) 2.4-F nitinol basket 63/72 (87.5)	3-F forceps 8/24 (33.3) BIGopsy forceps 7/24 (29.2) 2.4-F nitinol basket 9/24 (37.5)	LG 71/137 (51.8) HG 66/137 (48.2)	_	The quality of specimens from BIGopsy forceps was rated similarly to nitinol basket, and favoured over 3-F forceps specimens. No difference existed among the devices in the rate of acquiring a grade concordant biopsy.
Breda et al., 2019 [20]	85 (302 biopsies)	3-F forceps BIGopsy forceps 2.2-F nitinol basket	3-F forceps 164/219 (74.9) BIGopsy forceps 50/61 (81.9) 2.2-F nitinol basket 22/22 (100)	3-F forceps 55/66 (83.3) BIGopsy forceps 11/66 (16.7)	LG 75/140 (53.6) HG 50/140 (35.7) CIS 15/140 (10.7)	Tx 129/140 (92.1) Ta 11/140 (7.9)	BIGopsy forceps and the 2.2-F nitinol basket were superior to 3-F forceps for a successful histology characterisation. For papillary lesions, the basket biopsy provided larger specimens. For flat or sessile lesions, the BIGopsy forceps provided larger, deeper, and less distorted specimens than 3-F forceps.

CIS: carcinoma *in situ*; HG: high grade; LG: low grade.

Kleinmann et al. [19] evaluated the pathological result obtained in 504 URS-GB performed for suspicion of UTUC. The 2.4-F basket device proved to be superior to the 3-F cup forceps for disease confirmation (94% vs 63%, respectively; *P* < 0.001) and correct assessment of grade (93% vs 80%, respectively; *P* < 0.033).

Breda et al. [20] found BIGopsy forceps and the 2.2-F nitinol basket to be superior to 3-F forceps in obtaining an adequate specimen for the pathology examination of UTUC. In addition, for papillary lesions, the basket biopsy provided larger specimens in comparison to the other biopsy devices, while for flat or sessile lesions, the BIGopsy forceps provided larger, deeper, and less distorted specimens than the 3-F forceps.

In a multi-institutional retrospective study on 145 patients, Lama et al. [21] evaluated URS-GB using backloaded cup forceps, a nitinol basket, or standard cup forceps. The ability to distinguish low- and high-grade UTUC prior to RNU was statistically higher using the backloaded cup forceps (*P* = 0.02). The authors also reported that, compared with the standard cup forceps, the backloaded cup forceps showed a significantly higher subjective score for biopsy quality (*P* = 0.01), quality of basement membrane thickness (*P* = 0.02), and role of biopsy size in accurate diagnosis (*P* = 0.04). However, no subjective differences were noticed between the backloaded cup forceps and nitinol basket biopsies.

#### Case selection for conservative endoscopic treatment

The main factors that correlate with the oncological outcomes of URS treatment are pathological grade, tumour size, and tumour focality [2], although recent studies suggest that tumour burden plays a secondary role [22,23]. Therefore, risk stratification based on these factors is essential to limit the risk of under- and overtreatment, with current guidelines recommending elective conservative treatment only in the case of small (<2 cm), unifocal, low-grade disease [24]. In this context, endoscopic exploration of the UUT plays a key role in selecting those patients who are fit for KSS of UTUC, together with urinary cytology and cross-sectional imaging. Golan et al. [25], in their retrospective study on 116 patients who underwent d-URS for suspected UTUC on CTU, found that RNU was spared in 42% of cases, without reporting disease progression in any of those cases. Tsivian et al. [26] found a lower rate of RNU (89% vs 69%; *P* = 0.01) and misdiagnosis (15.5% vs 2.1%; *P* = 0.02) in patients evaluated with routine d-URS. Gallioli et al. [7] reported a change in the indication of elective treatment after d-URS in 37/76 (48.1%) cases (Table 3 [7,25,26]).

**Table 3.** Summary of published studies focussed on case selection for conservative endoscopic treatment

Reference	Patients, *n*	Purpose of the study	Results	*P*	Conclusion
Golan et al., 2015 [25]	116	Evaluation of diagnostic value of d-URS in cases suspected for UTUC and assessment of the impact of d-URS on the management of UTUC.	Positive urinary cytology PPV and NPV for UTUC: 47% and 58%. Ultrasound PPV and NPV for UTUC: 89% and 65%. CTU (filling defect) PPV and NPV for UTUC: 76% and 80%. CTU (wall thickening) PPV and NPV for UTUC: 67% and 90%. UTUC confirmed at URS: 29/38 (76%) patients with characteristic filling defect on CTU -> d-URS ruled out UTUC in 9 patients. According to tumour characteristics based on URS-GB result, 7/29 patients underwent conservative approach.	_	RNU was spared in (16/38) 42% of patients. In about half of those patients UTUC was ruled out and the others were managed endoscopically.
Tsivian et al., 2014 [26]	118	Comparing the results and outcomes in a group of patients who underwent routine d-URS (Group 2, *n* = 55) for suspected UTUC and a group who did not (Group 1, *n* = 63).	Group 1 pathology-confirmed UTUC: 49 (77.8%) Group 2 pathology-confirmed UTUC: 47 (85.5%) Group 1 RNUs performed: 55 (89%) Group 2 RNUs performed: 38 (69%) Group 1 misdiagnosis rate: 9/58 (15.5%) Group 2 misdiagnosis rate: 1/48 (2.1%)	0.347 0.011 0.021	Routine d-URS study for patients with suspected UTUC appeared to decrease the rate of RNU. Moreover, d-URS increased the diagnostic accuracy and reduced the rates of misdiagnosis of UTUC.
Gallioli et al., 2020 [7]	208	Analysis of the correspondence between imaging, d-URS and histology for UTUC.	URS was positive for UTUC in 107/115 (93%), 48/77 (62.3%), 15/27 (55.6%), 12/25 (48%) in positive, suspicious, unlikely and negative CTU, respectively. On cyto-histology, UTUC was confirmed in 164/182 (90.1%) cases. PPV on CTU was: 87.7% (121/138) for filling defect 65.6% (21/32) for stenosis 69.6% (64/92) for thickening 79.7% (59/74) for hydronephrosis	<0.001 0.04	A filling defect (*P* < 0.001) or hydronephrosis (*P* = 0.04) were associated with d-URS outcome. The indication of elective treatment changed after d-URS in 37/76 (48.1%) cases.

NPV: negative predictive value; PPV: positive predictive value.

Given its ability to consistently evaluate tumour dimension, focality, and growth pattern through direct visualisation, and to yield tumour grading via histopathology and *in situ* cytology, URS represents an essential tool for risk stratification and case selection for conservative endoscopic treatment.

#### Case selection for neoadjuvant chemotherapy

Several retrospective studies have shown the promising role of NAC in the treatment of high-risk locally advanced UTUC in comparison to RNU alone [24]. It must always be borne in mind that deterioration in renal function following RNU may render a patient ineligible for further cisplatin-based combination chemotherapy in 49% of cases [27], strengthening the role of NAC as opposed to adjuvant chemotherapy. However, the criteria for candidate selection remain unclear, although they commonly include high-grade pathology or locally advanced disease (cT2–T4N0M0) [28].

In this setting, URS can provide histopathological diagnosis, confirm the urothelial nature of the disease, and yield information about its grade, which has been shown to correlate with a higher stage and worse outcomes [29]. Furthermore, it helps candidate selection by detecting the presence of high-risk features such as high-grade tumour sessile growth patterns or large tumour burdens [30].

In the challenge to select those patients who will respond to NAC, the retrieval of a biopsy specimen could in the future provide a molecular fingerprint of the disease. It could also provide information regarding DNA repair genes, expression of tyrosine kinase receptors, immune checkpoint inhibitors molecular targets and regulation of apoptosis; this would allow the medical oncologist to select the best NAC or immunotherapy for patients in a truly tailored approach [31]. Table 4 [27,30,31] summarises the results of NAC for UTUC based on the included studies.

**Table 4.** Results of NAC for UTUC in the included studies

Reference	Patients, *n*	Purpose of the study	Results	*P*	Conclusion
Lane et al., 2010 [27]	336	Evaluate the eligibility to receive neoadjuvant or adjuvant CBCC in patients with UTUC.	Patients considered eligible to receive CBCC before RNU: 48% Patients considered eligible to receive CBCC after RNU: 22%	<0.001	Multimodal treatment strategies for UTUC should consider NAC, as few patients were eligible for adjuvant CBCC after RNU.
Porten et al., 2014 [30]	112	Comparison of the survival rates of patients with UTUC who received NAC before surgery (Group 1) with the rates among patients who did not (Group 2).	Group 1 patients: 31 (27.7%) Group 2 patients: 81 (72.3%) Group 1, 5-year CSS rate: 90.1% Group 2, 5-year CSS rate: 57.6% Group 1, 5-year OS rate: 80.2% Group 2, 5-year OS rate: 57.6%	<0.020 0.002	As NAC improved survival of patients with UTUC, patients with high-risk UTUC should be considered for NAC in view of the limited opportunity to administer effective CBCC after RNU.
Tse et al., 2019 [31]	_	Analysis of molecular predictors of complete response after NAC in UTUC and bladder tumours.	The authors analysed molecular markers to try to predict the response to CBCC including DNA repair genes (*ATM, RB1, FANCC, ERCC2, BRCA1*, and *ERCC1*), regulators of apoptosis (survivin, Bcl-xL, and emmprin), receptor tyrosine kinases (EGFR and erbB2), genes involved in cellular efflux (*MDR1* and *CTR1*), in addition to molecular subtypes (basal, luminal, and p53-like).	_	NAC offered advantages as patients have better renal function than after RNU. The use of biomarkers to stratify NAC administration based on predicted response could be a cost-effective option, although prospective validation was necessary.

CBCC: cisplatin-based combined chemotherapy; OS: overall survival.

#### New diagnostic technologies to increase diagnostic accuracy

Various new techniques have been developed to enhance diagnostic accuracy and risk stratification, including narrow-band imaging (NBI), the Storz professional imaging enhancement system (IMAGE1S), optical coherence tomography (OCT), and confocal laser endomicroscopy (CLE) (Table 5 [32–36]).

**Table 5.** Overview of optical/imaging techniques used during URS inspecting the UUT

Reference	Patients, *n*	Optical/imaging technique	Patient’s histology (*n*)	Imaging modality technical details	Sensitivity (%)	Specificity (%)	Conclusion
Traxer et al., 2011 [32]	27	NBI	UTUC (19) Benign (2) Invalid biopsy (6)	URF-V Olympus digital flexible ureteroscope with NBI system, 41 nm blue light and 540 nm green light.	_	_	NBI for UTUC was a valuable method because it considerably improved tumour detection rate by 22.7% compared with White light.
Emiliani et al., 2017 [33]	_	SpiesTM system	_	White light, Spectra A and B, Clara, Chroma, Clara + Chroma.	_	_	Clara and Clara + Chroma were ranked as the best Spies^TM^ modalities with better image quality.
Bus et al., 2016 [34]	26	OCT	UTUC (24) pTis-pTa (14) ≥pT1 (12)	C7-XR OCT system, 1300 nm longitudinal 54 mm and 360° trajectory taking 5.4 s.	Grading (86.7) Staging (91.7)	Grading (78.6) Staging (78.6)	OCT, using backscattered light instead of back reflected sound waves to produce cross-sectional images, had the potential to provide real-time information on grade and stage in UTUC.
Bui et al., 2015 [35]	14	CLE	UTUC (7) LG (4) HG (3) Benign (7)	0.85-mm diameter probe, with a depth of tissue penetration of 50 µm, field of view of 320 µm, and spatial resolution of 3.5 µm.	_	_	Optical biopsy using CLE in the UUT was feasible and provided real-time *in vivo* microscopy with sufficient resolution to distinguish between benign tissue and UC.
Breda et al., 2018 [36]	14	CLE	UTUC (12) LG (6) HG (5) CIS (1) Unknown (2)	Cellvizio system (Mauna Kea Technologies).	_	_	The authors found correspondence between the CLE images and the final histopathological results in 7/7 cases of low-grade UTUC (100%), in 5/6 cases of high-grade UTUC (83%), and in 1/1 case of CIS (100%).

CIS: carcinoma *in situ*; HG: high grade; LG: low grade.

Traxer et al. [32] reported a 22.7% increase in the tumour detection rate in the UUT when using NBI compared with white light. Emiliani et al. [33] reported CLARA and CLARA+CHROMA to offer better quality compared with white light and other IMAGE1S modalities.

OCT might enhance the staging accuracy of d-URS. Bus et al. [34] reported concordance of lesion staging with OCT and final histopathology in 83% of cases.

CLE provides real-time *in vivo* microscopy of tissue, allowing the distinction between benign tissue and UTUC [35]. Breda et al. [36] reported concordance between CLE images and final histopathological results in 100% and 83% of cases of high- and low-grade UTUC, respectively, and in one out of one case of carcinoma *in situ*.

### Cons

#### Risk of complications

During URS, lesions of the UUT can occur, ranging from mucosal abrasion to false passage, perforation and fluid extravasation, intussusception, and ureteric avulsion [37]. Bleeding is usually self-limited; nevertheless, life-threatening haemorrhagic complications and perirenal haematoma formation have been published as a result of forniceal or renal parenchyma rupture [38].

Among the early postoperative complications, UTI has been reported in 1–2.6% of cases, with severe sepsis in 0.06–0.3% [39,40], the latter probably linked to the development of high intrarenal pressures during the procedure [41]. Local oedema, ureteric spasm, or blood clot can lead to acute obstruction of the UUT and renal colic. VUR may occur after URS, but the use of small-calibre flexible and semi-rigid ureteroscopes has obviated ureteric dilatation in most cases and has likely reduced the incidence of reflux overall [37].

Ureteric strictures can occur as a late postoperative complication subsequent to ureteric lesions. Although the incidence of strictures has been reported to be <1% [39,40], the use of post-ureteroscopic lesion scores has revealed the incidence to be considerably higher [42,43] (Table 6 [38–40,42,43]).

**Table 6.** Intra- and postoperative complications rate reported in the studies included in our review during operative URS

Reference	Patients, *n*	Intraoperative complications reported	*N* (%)	Postoperative complications reported	*n/N* (%)
Traxer et al., 2013 [42]	359	Ureteric wall injuries resulting from insertion of a UAS Grade 0 (no lesion) and 1 (mucosal erosion without smooth muscle injury) Grade 2 (involvement of smooth muscle without adventitial) Grade 3 (adventitial perforation without ureteric avulsion) Grade 4 (total ureteric avulsion)	311 (86.6) 37 (10.3) 11 (3.1) 0 (0)	Pyelonephritis Pyelonephritis	6/311 (1.93) 4/48 (8.33)
Schoenthaler et al., 2014 [43]	100	Ureteral trauma (according to PULS) Grade 0 (insignificant lesion) Grade 1 (superficial mucosal lesion) Grade 2 (submucosal lesion) and 3 (perforation with less than 50% partial transection)	43 (43) 44 (44) 13 (13)	_	_
Whitehurst et al., 2017 [38]	8929	_		Perirenal haematoma	40 (0.45)
Somani et al., 2017 [39]	11,885	Bleeding Perforation Failed access Conversion Other Mucosal injury Infection	167 (1.41) 124 (1.05) 198 (1.67) 19 (0.16) 177 (1.49) 15 (0.13) 8 (0.07)	Bleeding Fever UTI Sepsis Pain Urinary retention Stent misplacement Other	54 (0.45) 204 (1.72) 113 (0.95) 36 (0.30) 39 (0.33) 13 (0.11) 12 (0.10) 39 (0.33)
Baş et al., 2017 [40]	1395	Mucosal injury Malfunctioning or breakage of instruments Bleeding Perforation Severely bleeding	37 (2.35) 2 (0.13) 40 (2.55) 5 (0.32) 9 (0.57)	Fever Bleeding UTI Stent migration Ureteric stricture Other	84 (5.35) 25 (1.59) 42 (2.67) 11 (0.70) 2 (0.13) 12 (0.76)

PULS: post-ureteroscopic lesion scale; UAS: ureteric access sheath.

#### Cancer dissemination

The potential role of URS in cancer dissemination was initially explored by Kulp et al. [44] in 1994, who reported on a series of 13 patients who underwent URS prior to RNU. In the surgical specimens, no tumour cells were noted in vascular or lymphatic spaces. Conversely, Lim et al. [45] described a case of suspicious lymphatic invasion that the authors attributed to high intrarenal pressures during URS.

Comparing patients with and without d-URS, Hendin et al. [46] found no significant differences in overall 5-year survival (87% vs 76%,) or metastasis-free survival (MFS) (67% vs 71%). Ishikawa et al. [47], in a multi-institutional study of 208 patients, found the 5-year cancer-specific survival (CSS) to be comparable in the d-URS and control groups (88.3% vs 78.1%). Gurbuz et al. [48] explored the recurrence-free survival (RFS) of patients with and without d-URS prior to RNU and found comparable results at 5-year follow-up (72% vs 69%; *P* = 0.17).

Guo et al. [49] published a meta-analysis that confirmed the above findings. Despite the paucity of data and the retrospective design of all the eight studies included, the authors concluded from that meta-analysis that URS is not associated with overall survival, MFS, or RFS. Table 7 [46–48] summarises the results of the studies concerning cancer dissemination included in the review.

**Table 7.** Summary of included studies analysing cancer dissemination

Reference	Patients, *n*	Treatments, (*n*)	Surgical approach	Survival analysis or difference between groups analysed	*P*
Hendin et al., 1994 [46]	96	RNU or DU (-URS) (48) URS + RNU or DU (48)	RNU or DU with bladder cuff	Metastases developed in RNU or DU (-URS) group: 9 (18.8%) Metastases developed in URS + RNU or DU group: 6 (12.5%) Death for metastases in RNU or DU (-URS) group: 5 (10.4%) Death for metastases in URS + RNU or DU group: 5 (10.4%) 5-year MFS in RNU or DU (-URS) group: 71% 5-year MFS in URS + RNU or DU group: 67% 5-year OS in RNU or DU (-URS) group: 76% 5-year OS in URS + RNU or DU group: 87%	0.58 1.00 0.25 0.75
Ishikawa et al., 2010 [47]	208	RNU (-URS) (153) URS + RNU (55)	RNU (165 open, 43 laparoscopic)	2-year bladder RFS in RNU (-URS) group: 58.7% 2-year bladder RFS in URS + RNU group: 60% 5-year CSS in RNU (-URS) group: 78.1% 5-year CSS in URS + RNU group: 88.3%	0.972 0.069
Gurbuz et al., 2011 [48]	1268	ETA + RNU (175) RNU without previous ETA (1093)	RNU (970 open, 298 laparoscopic)	ETA + RNU 5-year RFS and CSS rates: 72% and 77% RNU without previous ETA 5-year RFS and CSS rates: 69% and 73%	0.171 0.365

DU: distal ureterectomy; ETA: endoscopic tumour ablation; OS: overall survival.

#### Bladder recurrence

In recent years, the risk of intravesical recurrence (IVR) after URS prior to RNU has been addressed. The hypothesis is that the manipulation of the ureteroscope and the irrigation back-flow may increase the risk of or seeding [45]. Audenet et al. [50] showed that the majority of bladder tumours following RNU are clonally related, supporting the hypothesis that IVR is caused by neoplastic cell implantation rather than being a second primary tumour.

Two meta-analyses have summarised the current evidence. Marchioni et al. [51] found an IVR rate of 39.2–60.7% and 16.7–46% in patients with and without URS prior to RNU, respectively. The pooled analysis found a significant association between URS and IVR (hazard ratio [HR] 1.56; *P* < 0.001). Guo et al. [49] similarly found that IVR rates were lower in patients without a history of bladder cancer who underwent URS (HR 1.81). The main limitations of these two meta-analyses are the retrospective design of all the included studies, the lack of data about the URS, and the bladder cuff management technique [52] with no postoperative administration of mitomycin C [53].

Conversely, Ishikawa et al. [47] found that IVR at 2-year follow-up was comparable (60% vs 58.7%) in patients who underwent URS prior to RNU and controls. Similar results were reported by Lee et al. [54].

Sung et al. [55] found that the interval between URS and RNU does not seem to affect the IVR rate, while Lee et al. [56], reported that patients who underwent URS and RNU in a single session had an IVR comparable to that in a non-URS group.

Yoo et al. [57] found the IVR rate to be significantly higher in patients with renal pelvic (but not ureteric) tumours who underwent URS-GB prior to RNU (60.4%; HR 2.06; *P* = 0.01). A possible explanation is that the endoscopic manipulation could have increased the shedding of cancer cells from the renal pelvis into the bladder. Table 8 [47,55–58] summarises the results of bladder recurrence for UTUC based on the included studies.

**Table 8.** Overview of IVR after URS prior to RNU in the included studies

Reference	Patients, *n*	Treatments (*n*)	IVR rate (%)	Median/mean follow-up, months	Bladder cuffing	NAC/adjuvant therapy	pT stage: %	Grade (%)	Conclusion
Ishikawa et al., 2010 [47]	208	RNU (-URS) (153) URS + RNU (55)	RNU (-URS) group (41.3) URS + RNU group (40)	44	Yes	No	Ta–CIS: 19.7 T1: 28.4 T2: 21.2 T3: 26.9 T4: 3.8	LG (68.3) HG (31.7)	The 2-year bladder RFS rate between the two groups was not significantly different (*P* = 0.972).
Lee et al., 2018 [54]	502	RNU (-URS) (296) URS + RNU (206)	138 patients (27.5)	76	Yes	No	Ta–CIS: 14.1 T1: 26.1 T2: 25.3 T3: 28.7 T4: 5.8	LG (22.1) HG (77.9)	URS-GB performed before RNU did not appear to be a prognostic factor of IVR (*P* = 0.609).
Sung et al., 2015 [55]	630	RNU (-URS) (348) URS + RNU (282)	RNU (-URS) group (36.4) URS + RNU group (57.4)	34.3	Yes	128 patients (20.3%)	Ta: 17.3 T1: 24.3 T2: 16.3 T3–T4: 42.1	I–II (55.1) III (44.9)	URS was associated with increased IVR rate following RNU. Prior bladder tumour history, multifocal UTUC, and extravesical cuff excision were also predictors of IVR.
Lee et al., 2016 [56]	104	RNU (-URS) (30) URS + RNU (74)	RNU (-URS) group (16.7) URS + RNU I session group (24.2) URS + RNU II session group (51.2)	37.0 29.6 36.4	_	No	T ≤ 1: 38.8 T2: 45.6 T3: 15.6	LG (24) HG (76)	Delay of RNU after URS significantly increased IVR in patients with UTUC. Performing URS and RNU in one session was preferable.
Yoo et al., 2017 [57]	387	RNU (-URS) (318) URS + RNU (69)	RNU (-URS) group in RPT (37.8) RNU + URS group in RPT (60.4) RNU (-URS) group in UT (54.2) RNU + URS group in UT (63.3)	62	Yes	No	Ta–T1: 53.5 T2: 17.6 T3–T4: 28.9	LG (50.6) HG (49.4)	URS-GB was a significant risk factor for IVR in patients with RPT but not for UT. So that URS-GB should only be performed for suspected RPT.

*CIS: carcinoma *in situ*; ETA: endoscopic tumour ablation; HG: high grade; LG: low grade; RPT: renal pelvic tumour; UT: ureteric tumour.

#### Delay in radical treatment

One of the main criticisms regarding the systematic implementation of URS for UTUC is that it may lead to a delay in radical surgical treatment. It has been shown that a delay in bladder cancer treatment is associated with a higher pathological stage, and the window of time between diagnosis and cystectomy should not exceed 12 weeks [58]. This threshold does not apply to UTUC [59,60]. Nison et al. [61] reported on a significant delay in surgical treatment following URS (median 79.5 days) compared with a non-URS group (median 44.5 days; *P* = 0.04). However, the 5-year CSS, MFS, and RFS were comparable between the groups. Boorjian et al. [62] retrospectively compared the results in 121 patients who underwent RNU without URS (*n* = 34), with URS-GB (*n* = 75), or with URS + laser ablation (*n* = 12). They found no significant difference in postoperative disease status between the three groups.

Gurbuz et al. [48], in a multi-institutional retrospective study, confirmed that endoscopic ablation prior to RNU is not associated with poorer CSS and disease-free survival. The study demonstrated patient selection for laser ablation to be the key in guaranteeing good oncological outcomes even after endoscopic management failure. Table 9 [48,62–63] summarises the results of the studies concerning delay in radical treatment included in the review.

**Table 9.** Overview of delay in radical treatment and reported outcomes

Reference	Patients, *n*	Treatments (*n*)	Survival analysis or difference between groups analysed	*P*	Conclusion
Nison et al., 2013 [61]	512	RNU (-URS) (342) URS + RNU (170)	Median treatment time in RNU (-URS) group: 44.5 days Median treatment time in URS + RNU group: 79.5 days 5-year CSS did not differ between RNU (-URS) and URS + RNU group 5-year RFS did not differ between RNU (-URS) and URS + RNU group 5-year MFS did not differ between RNU (-URS) and URS + RNU group	0.04 0.23 0.89 0.35	Despite the increased time to radical surgery, URS could be systematically performed to refine the therapeutic strategy without significantly affecting oncological outcomes.
Boorjian et al., 2005 [62]	121	RNU (-URS) (34) URS + RNU (75) URS + ETA + RNU (12)	Median FU in RNU (-URS) group: 38.7 months Median FU in URS + RNU group: 40.1 months Median FU in URS + ETA + RNU group: 37.2 months 23/34 patients of the RNU (-URS) group had Ta or T1 tumours 49/75 patients RNU + URS group had Ta or T1 tumours 8/12 patients of the group URS + ETA + RNU had Ta or T1 tumours 29/34 (85.3%) patients of the RNU (-URS) group had no evidence of disease at last FU 61/75 (81.3%) patients of the RNU + URS group had no evidence of disease at last FU 10/12 (83.3%) patients of the URS + ETA + RNU had no evidence of disease at last FU	0.73 0.99 0.16	URS and/or ETA were not associated with an increased frequency of adverse tumour pathological features, and neither the tumour grade nor stage at RNU was significantly different among the groups. URS and/or laser ETA before RNU did not significantly affect the postoperative outcomes.
Gurbuz et al., 2011 [48]	1268	ETA + RNU (175) RNU without previous ETA (1093)	ETA + RNU 5-year RFS and CSS rates: 72% and 77% RNU without previous ETA 5-year RFS and CSS rates: 69% and 73%	0.171 0.365	In selected patients, ETA did not adversely affect the recurrence and survival after subsequent RNU for UTUC.

ETA: endoscopic tumour ablation; FU: follow-up.

## Conclusions

A d-URS is an extremely valuable tool in cases of suspected UTUC, especially when the diagnosis is equivocal or when KSS can be considered. Although nowadays URS can be considered a safe procedure, it is not without risks, some of which can endanger the patient’s life. Risks can be related to the presence of neoplastic disease, which translates into the potential risk of bladder recurrence, cancer dissemination, and/or delays in radical treatment. Some technical devices or precautions can aid in obtaining correct information, above all concerning the grade of the disease. The latest techniques such as NBI, IMAGE1S, CLE, and OCT can also provide information of value in optimising patient selection for KSS. NAC could represent an ‘ace up our sleeve’, but accurate staging and grading remain crucial for appropriate therapeutic decision making, and d-URS can be of decisive importance for this purpose.
